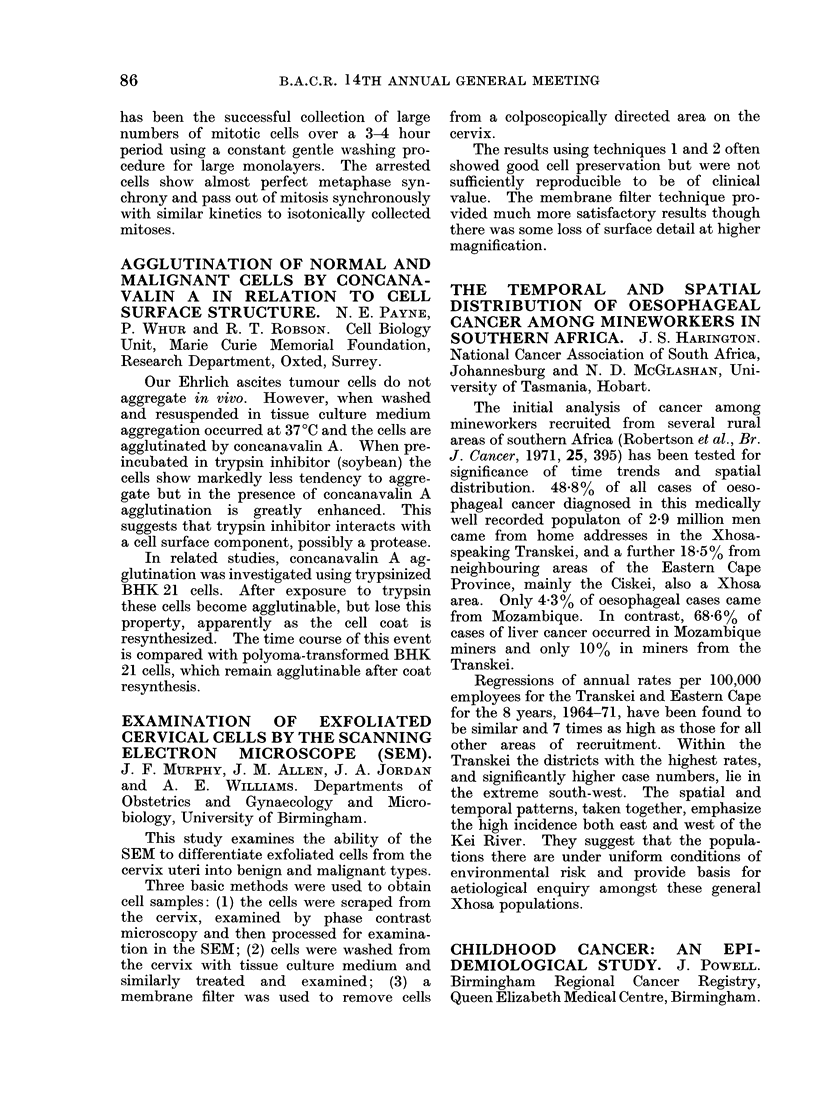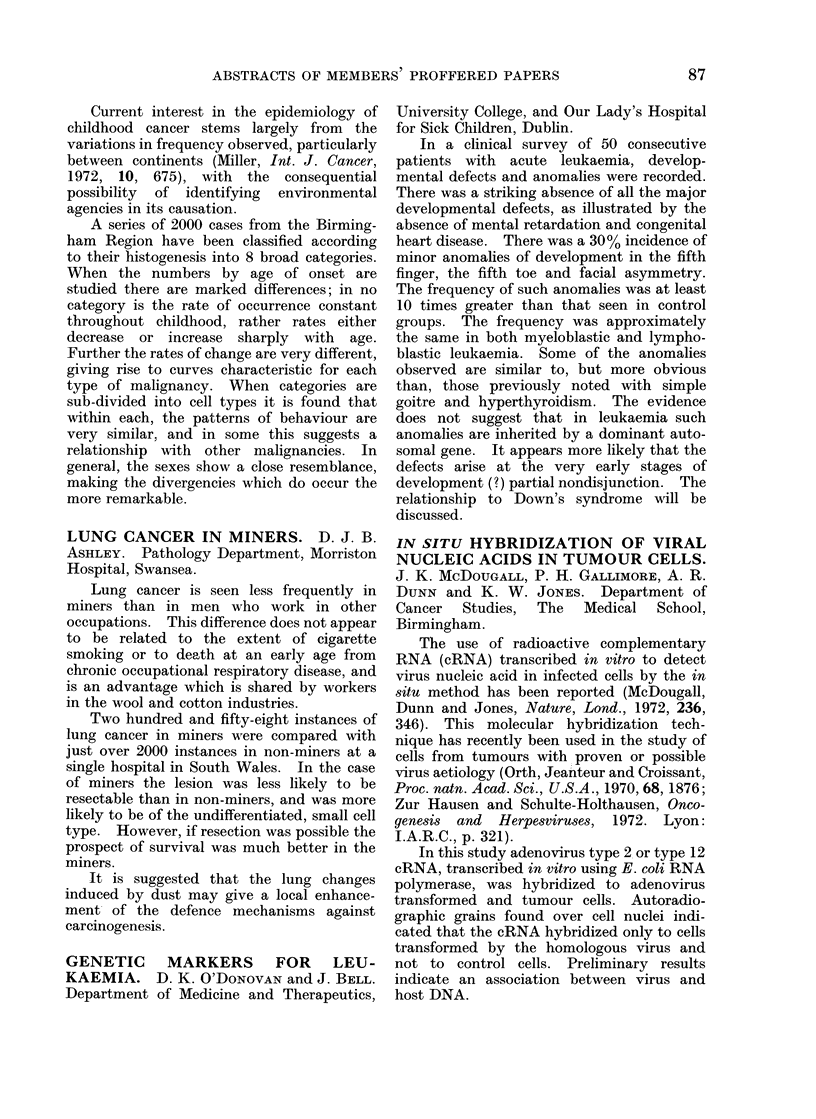# Childhood cancer: an epidemiological study.

**DOI:** 10.1038/bjc.1973.110

**Published:** 1973-07

**Authors:** J. Powell


**CHILDHOOD CANCER: AN EPIDEMIOLOGICAL STUDY.** J. POWELL. Birmingham Regional Cancer Registry, Queen Elizabeth Medical Centre, Birmingham.

Current interest in the epidemiology of childhood cancer stems largely from the variations in frequency observed, particularly between continents (Miller, *Int. J. Cancer*, 1972, **10**, 675), with the consequential possibility of identifying environmental agencies in its causation.

A series of 2000 cases from the Birmingham Region have been classified according to their histogenesis into 8 broad categories. When the numbers by age of onset are studied there are marked differences; in no category is the rate of occurrence constant throughout childhood, rather rates either decrease or increase sharply with age. Further the rates of change are very different, giving rise to curves characteristic for each type of malignancy. When categories are sub-divided into cell types it is found that within each, the patterns of behaviour are very similar, and in some this suggests a relationship with other malignancies. In general, the sexes show a close resemblance, making the divergencies which do occur the more remarkable.